# A Distal ABA Responsive Element in *AtNCED3* Promoter Is Required for Positive Feedback Regulation of ABA Biosynthesis in Arabidopsis

**DOI:** 10.1371/journal.pone.0087283

**Published:** 2014-01-27

**Authors:** Yan-Zhuo Yang, Bao-Cai Tan

**Affiliations:** Institute of Plant Molecular Biology and Agricultural Biotechnology, State Key Lab of Agrobiotechnology, School of Life Sciences, The Chinese University of Hong Kong, Shatin, N.T., Hong Kong, China; University of Delhi South Campus, India

## Abstract

The plant hormone abscisic acid (ABA) plays a crucial role in plant development and responses to abiotic stresses. Recent studies indicate that a positive feedback regulation by ABA exists in ABA biosynthesis in plants under dehydration stress. To understand the molecular basis of this regulation, we analyzed the *cis*-elements of the *AtNCED3* promoter in Arabidopsis. *AtNCED3* encodes the first committed and highly regulated dioxygenase in the ABA biosynthetic pathway. Through delineated and mutagenesis analyses in stable-transformed Arabidopsis, we revealed that a distal ABA responsive element (ABRE: GGCACGTG, -2372 to -2364 bp) is required for ABA-induced *AtNCED3* expression. By analyzing the *AtNCED3* expression in ABRE binding protein ABF3 over-expression transgenic plants and knock-out mutants, we provide evidence that the ABA feedback regulation of *AtNCED3* expression is not mediated by ABF3.

## Introduction

Abscisic acid (ABA) plays a critical role in several plant developmental processes including inhibition of seed germination and promotion of seed dormancy [1], and mediates plant responses to various environmental stresses, particularly drought and salinity [2], [3]. Under drought or salinity stress, plants quickly synthesize and accumulate up to 40-folds of ABA [4], which facilitates stomatal closure and expression of ABA responsive genes to protect plants from further water loss and damage. For this reason, understanding the mechanism by which stress induces ABA biosynthesis will provide insights to engineering stress tolerant crops.

ABA is synthesized by oxidative cleavage of epoxy-carotenoids in plants [4]. Key steps in the pathway have been identified in various plant species [5]. The epoxidation of zeaxanthin and antheraxanthin to form violaxanthin and neoxanthin is catalyzed by zeaxanthin epoxidase (ZEP/AtABA1) [6], [7]. The products are isomerized to produce 9-*cis* isomers which are cleaved by nine-*cis*-epoxycarotenoid dioxygenase (NCED) to form xanthoxin [8]–[10]. The later is subsequently converted to ABA by two oxidases, a short-chain dehydrogenase/reductase (SDR) and aldehyde oxidase 3 (AAO3) [11]–[13]. The activity of the AAO3 enzyme requires a sulfurated molybdenum cofactor (MoCo), which is converted from the desulfo- to the sulfo-form by the MoCo sulfurase ABA3 [14], [15]. Although the pathway is clear, the regulation of ABA synthesis remains poorly understood. Evidence indicates that the oxidative cleavage of epoxy-carotenoids by NCED is the first committed, highly regulated, step in ABA biosynthesis [9], [10], [16], [17], and that the accumulation of ABA is the result of *de novo* expression of *NCED*s in response to various stresses [9], [10], [16]. In Arabidopsis, loss of *AtNCED3* compromises ABA accumulation under drought stress, causing wilty plants [17]. Conversely, over-expression of *AtNCED3* in Arabidopsis and *LeNCED1* in tomato (*Lycopersicon esculentum*) enhances endogenous ABA accumulation and increases dehydration resistance [17], [18].

Biosynthetic pathways are frequently feedback-regulated by end-products. Plant hormones show both negative and positive regulations. Bioactive gibberellins (GA) negatively regulate GA biosynthesis by curtaining the transcription of GA20ox and GA3ox [19], [20]. Ethylene can both positively and negatively regulate ethylene biosynthesis by up regulating *LeACS2* and *LeACS4* and down regulating *LeACS6* transcription during fruit ripening in tomato [21]. Recent evidence indicates that a positive feedback regulation exists also in ABA biosynthesis. Indeed, exogenous ABA significantly enhanced the expression of *ABA1*, *AAO3* and *ABA3* and *AtNCED3* was induced by ABA in different Arabidopsis accessions [7], [12], [14], [22]. Furthermore, in the ABA deficient mutants *aba1* and *aba3*, the transcript of all the inducible ABA biosynthetic genes under stress were significantly lower than those in the wild-type [7], [14]. In addition, *AtNCED3* transcript were dramatically reduced in ABA insensitive mutants *snrk2.2/2.3/2.6* and *pyr1;pyl1;pyl2;pyl4* in comparison to the wild-type under ABA treatment [23], [24]. Taken together, these results strongly demonstrate that a positive feedback regulation exists in ABA biosynthesis, which contributes to ABA accumulation under drought stress.

Many ABA responsive genes contain conserved ABA responsive elements (ABRE, PyACGTGG/TC) in their promoter regions [25]. ABREs can be recognized by ABRE binding proteins/factors (AREBs/ABFs), which are a family of basic leucine zipper (bZIP) transcription factors [26], [27]. In general, a single copy of ABRE is not sufficient for ABA responsive gene expression; it requires two ABREs or one ABRE coupled with a coupling element (CE) [28]–[30]. Two ABREs are necessary for ABA-responsive expression of *RD29B* in Arabidopsis [27]. One ABRE and one CE are necessary for ABA induction of VP1 expression in maize (*Zea mays*) [31]. Similar case is also found in *HVA22* from barley (*Hordeum vulgare*) [22].

To understand the regulation of ABA biosynthesis, it is necessary to identify the *cis*-elements in the *AtNCED3* promoter that mediate the positive feedback regulation. In this study, we mapped the ABA responsive elements by deletion and mutation analysis in transgenic Arabidopsis. The results indicate that a distal ABRE (GGCACGTG) plays a crucial role in conferring ABA responsive expression of *AtNCED3*. This element shares conserved core sequence with drought induced *NCED* promoters from other species. We further show the evidence that ABF3, a known ABRE binding factor, does not regulate the expression of *AtNCED3*.

## Materials and Methods

### Plant materials, growth conditions and ABA treatment

Arabidopsis ecotype Columbia (Col-0) was used and *abf3* mutant (SALK_096965) was kindly provided by Prof. Qi Xie (Institute of Genetics and Developmental Biology, Chinese Academy of Science, China). Seeds were sterilized, sown on 1/2 MS [32] plates, and subjected to cold treatment for 3 days. The plates were transferred to a growth chamber with 16-h-light (PPFD  =  100 µmol m^−2^s^−1^) /8-h-dark cycle at 22°C for two weeks. Two-week-old seedlings were used for treatment or grown on soil at 22°C with 16 h of light daily.

For ABA treatment, 100 µM ABA in water was sprayed on leaves of two-week-old seedlings, or with water in the case of the controls, and the plants were incubated at 22°C for 5 h.

### Histochemical GUS staining and quantitative measurement of GUS activity

Histochemical GUS staining was performed as described previously [33]. 10 independent lines and at least 10 individual plants from each line were analyzed. Briefly, tissues were incubated in staining solution (0.5 mM potassium ferrocyanide, 0.5 mM potassium ferricyanide, 50 mM sodium phosphate buffer, pH 7.0, 0.1% Triton X-100, and 1 mM X-Gluc) at 37°C for 16h. Stained tissues were bleached in 70% ethanol for 16h.

Measurement of GUS activity in whole seedling was carried out by the method of Jefferson et al [34]. 10 independent lines and 10 individual plants from each line were analyzed. The fluorescence was measured by an Infinite M1000 microplatereader (TECAN Group Ltd). Protein concentration was determined by Protein assay reagent (Bio-Rad).

### Sequence analysis

The PlantCARE (http://bioinformatics.psb.ugent.be/webtools/plantcare/html/) database was used to analyze the promoter sequence of *AtNCED3*. Sequence logos were created online using the Weblogo resource (http://weblogo.berkeley.edu/).

### Quantitative RT-PCR analysis of gene expression

Total RNA was purified using Qiagen Plant RNeasy kit according to the manufacturer’s instructions and then treated with DNase I (New England Biolabs) to eliminate genomic DNA contamination. Reverse transcription reactions were performed using 1 µg of total RNA by SuperScriptII reverse Transcriptase (Invitrogen). Quantitative RT-PCR was performed in the iQ5 real-time PCR detection system using iQ SYBR Green Supermix (Bio-Rad). Each experiment was replicated three times. To quantify the copy number of each RNA, the Ct (threshold cycle) value was compared with a standard curve. The amplified DNA fragment was purified, quantified spectrophotometrically to generate a standard curve for each gene. All the transcripts were normalized as copy number per ng of total RNA. Sequences of primers are listed in Table S1.

### Plasmid construction and plant transformation

We made five constructs to identify ABA responsive region in –2664 bp *AtNCED3* promoter. Five DNA fragments containing 5′-deletion *AtNCED3* promoter regions (–2664, –2018, –1470, –774 or –349 to –4 bp) were amplified by PCR with forward primers (N3-PDF1, N3-PDF2, N3-PDF3, N3-PDF4 or N3-PDF5) and reverse primer (N3-PDR1) using Phusion DNA polymerase (New England Biolabs). PCR fragments were ligated into pENTR D/Topo (Invitrogen), and then recombined into *pGWB3* vector using Gateway LR Clonase II enzyme mix kit (Invitrogen).

Four constructs were designed to further confirm that the region between –2664 and –2018 bp contains ABA responsive *cis*-element. Two 3′-deletion fragments (–2664 to –2018 bp and –2664 to –1470 bp) were amplified by forward primer (N3-PDF1) and reverse primers (N3-FR1 or N3-FR2). The CaMV 35S minimal promoter (–46 to +8 bp) and –349 bp *AtNCED3* promoter (–349 to –4 bp) were amplified with 35SM-F1, 35SM-R1 and N3-PDF5, N3-PDR1, respectively. –2664 to –2018 bp and –2664 to –1470 bp regions were ligated with CaMV 35S minimal promoter or –349 bp *AtNCED3* promoter by overlap extension PCR (OE-PCR) as described by Wurch et al [35]. Briefly, –2664 to –2018 bp region was ligated with CaMV 35S minimal promoter or –349 bp *AtNCED3* promoter by OE-PCR with complementary primers (S35S-F, S35S-R and SN3-F, SN3-R). –2664 to –1470 bp region was ligated with CaMV 35S minimal promoter or –349 bp *AtNCED3* promoter by OE-PCR with complementary primers (L35S-F, L35S-R and LN3-F, LN3-R). The resultant fragments were ligated into pENTR D/Topo, and then introduced into *pGWB3*.

To narrow down ABA responsive *cis*-element region between the –2664 to –2018 bp region of *AtNCED3* promoter, additional 5′-deletion constructs (*np6*-*np9*) were generated. *AtNCED3* promoter regions (–2414, –2327, –2214 or –2092 to –4 bp) were amplified with forward primers (N3-PDF6, N3-PDF7, N3-PDF8 or N3-PDF9) and reverse primer (N3-PDR1) using Phusion DNA polymerase (NEB). PCR fragments were ligated into pENTR D/Topo (Invitrogen), and then recombined into *pGWB3* vector using Gateway LR Clonase II enzyme mix kit (Invitrogen).

We designed constructs with mutation of ABRE base substitution to investigate the effect of ABRE in -2664 bp *AtNCED3* promoter. The mutated promoter was generated with complementary primers (mABRE-F and mABRE-R) by OE-PCR. The resultant fragment was ligated into pENTR D/Topo, and then introduced into *pGWB3*.

To create three repeats of 87 bp region or mutated 87 bp region followed by the –349 bp *AtNCED3* promoter or CaMV 35S minimal promoter, firstly, 87 bp region or mutated 87 bp region were amplified by complementary primers (R87R-F, R87R-R), and then generated three tandem repeats of each fragment by OE-PCR. Three tandem repeats of 87 bp region and mutated 87 bp region was ligated with CaMV 35S minimal promoter or –349 bp promoter with complementary primers (R87S-F, R87S-R and R87N3-F, R87N3-R). The fused fragments were ligated into pENTR D/Topo, and then recombined into *pGWB3*.

The open reading frame of ABF3 was amplified using Phusion DNA polymerase and primers (ABF3-EF and ABF3-ER) to generate overexpression vector. The PCR product was introduced into pENTR D/Topo, and then recombined into *pGWB2*. The information of primers is listed in Table S1.

Arabidopsis transformation was carried out using floral-dip method as described previously [36]. T1 generation transgenic lines were selected on 1/2 MS plates containing hygromycin (final concentration of 25 mg/l). For each construct, more than 10 independent lines with a single insertion locus were selected based on the segregation of seedlings (3:1) that were resistant to hygromycin (final concentration of 25 mg/l) in T2 generation. T_2_ plants for a single insertion locus were used in histochemical GUS staining and quantitative measurement of GUS activity.

## Results

### The 2664 bp region of *AtNCED3* promoter from the translation start site possesses various regulatory elements, including ABA responsive *cis*-element

**Figure 1:**
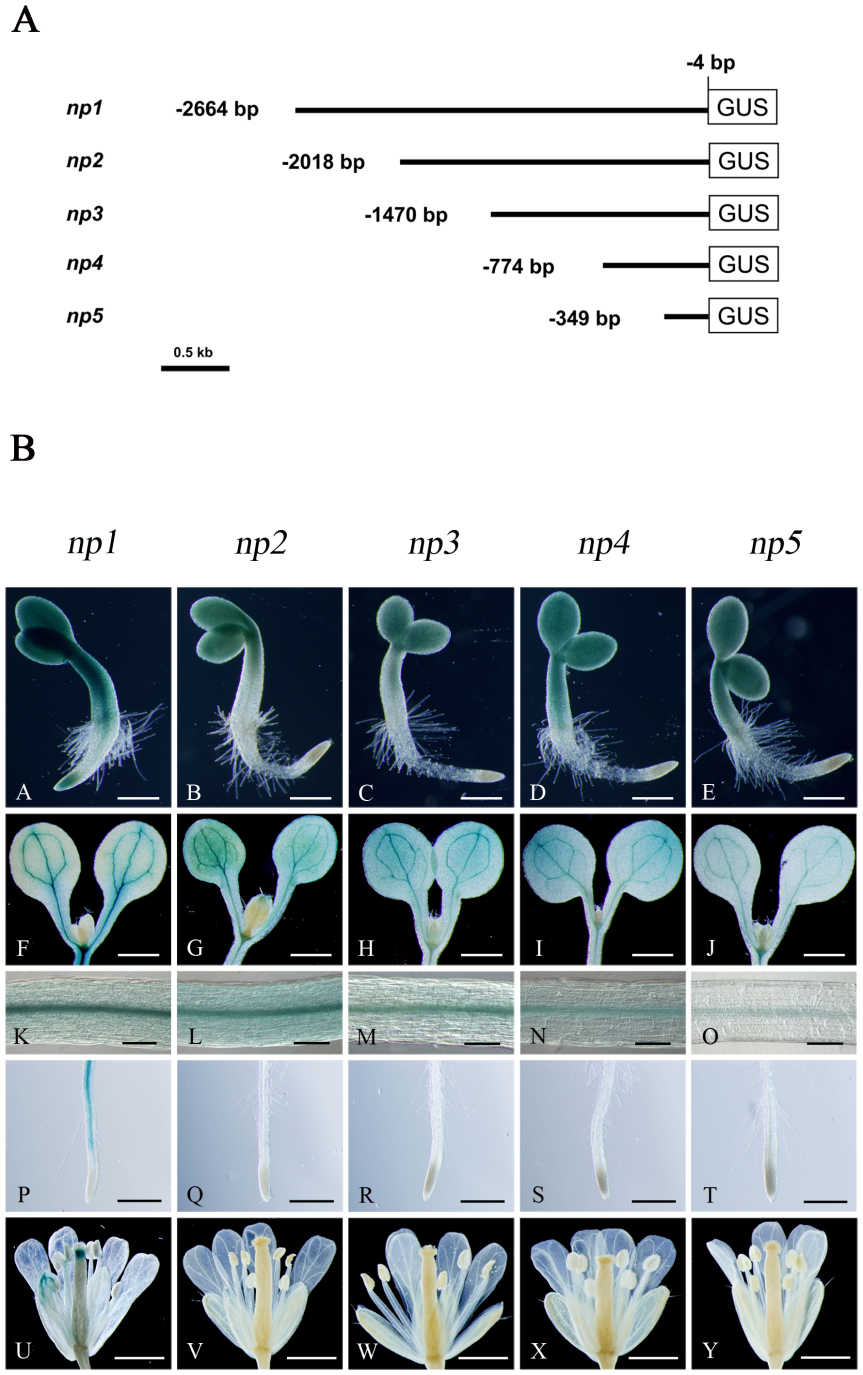
The expression pattern of transgenic plants harboring different 5′-deletion regions of *AtNCED3* promoter-GUS constructs. (A) The different 5’-deletion constructs of the *AtNCED3* promoter-GUS. The *AtNCED3* promoter regions between –2664 to –4 bp, –2018 to –4 bp, –1470 to –4 bp, –774 to –4 bp or –349 to –4 bp are cloned upstream of GUS coding region. (B) Histochemical analysis of GUS expression in transgenic plants carrying different lengths promoter. 10 independent lines and 10 individual plants from each line were analyzed by histochemical GUS staining. A to E: 36-h-old seedlings after germination (scale bars: 200 µm); F to J: cotyledon of 5-day-old seedlings (scale bars: 1 mm); K to O: section of hypocotyl of 5-day-old seedlings (scale bars: 220 µm); P to T: root (scale bars: 500 µm); U to Y: flower (scale bars: 1 mm).

We previously described that transgenic Arabidopsis expressing the *β-glucuronidase* (GUS) gene under the control of a 1.5 kb-long region of the *AtNCED3* promoter lacked GUS expression in response to dehydration stress [33]. In opposite, a 1.0 kb *AtNCED3* promoter driven firefly luciferase (*LUC*) in transgenic Arabidopsis has been reported to respond to osmotic stress [37], [38]. To analyze *AtNCED3* promoter, we developed in Arabidopsis a reporter assay taking into consideration various regions of the *AtNCED3* promoter ranging from 2.7 kb and 0.3 kb upstream of translation start site. The A of the ATG codon at the translational site was defined as +1. We generated five constructs that contained the *AtNCED3* promoter region between –2664 to –4 bp, –2018 to –4 bp, –1470 to –4 bp, –774 to –4 bp or –349 to –4 bp fused with GUS gene (Figure 1A, *np1*-*np5*). Transgenic Arabidopsis plants were generated, as named after the constructs. At least 10 independent plants from each individual transgenic line were analyzed by histochemical GUS staining. Three developmental stages were observed: 36 h after germination, 5-day-old seedlings and the reproductive stage.

In early seedling developmental stage, GUS activity was found in cotyledons of transgenic plants containing any length promoter (Figure1B, A-E), whereas GUS expression was only observed in radicle tips of *np1* transgenic plants (Figure1B, A). In 5-day-old seedlings, GUS expression was observed in vascular tissue of cotyledons and stems of transgenic plants containing any promoter fragment (Figure1B, F-O), however, the expression in maturation zone of root was only found in *np1* transgenic plants (Figure1B, P). During the reproductive stage, only *np1* transgenic plants showed GUS activity in styles and vascular tissue of sepals (Figure1B, U). These results indicate that the 2664 bp of *AtNCED3* promoter from translation start site possesses various regulatory elements involving in tissue specific expression of *AtNCED3*.

**Figure 2:**
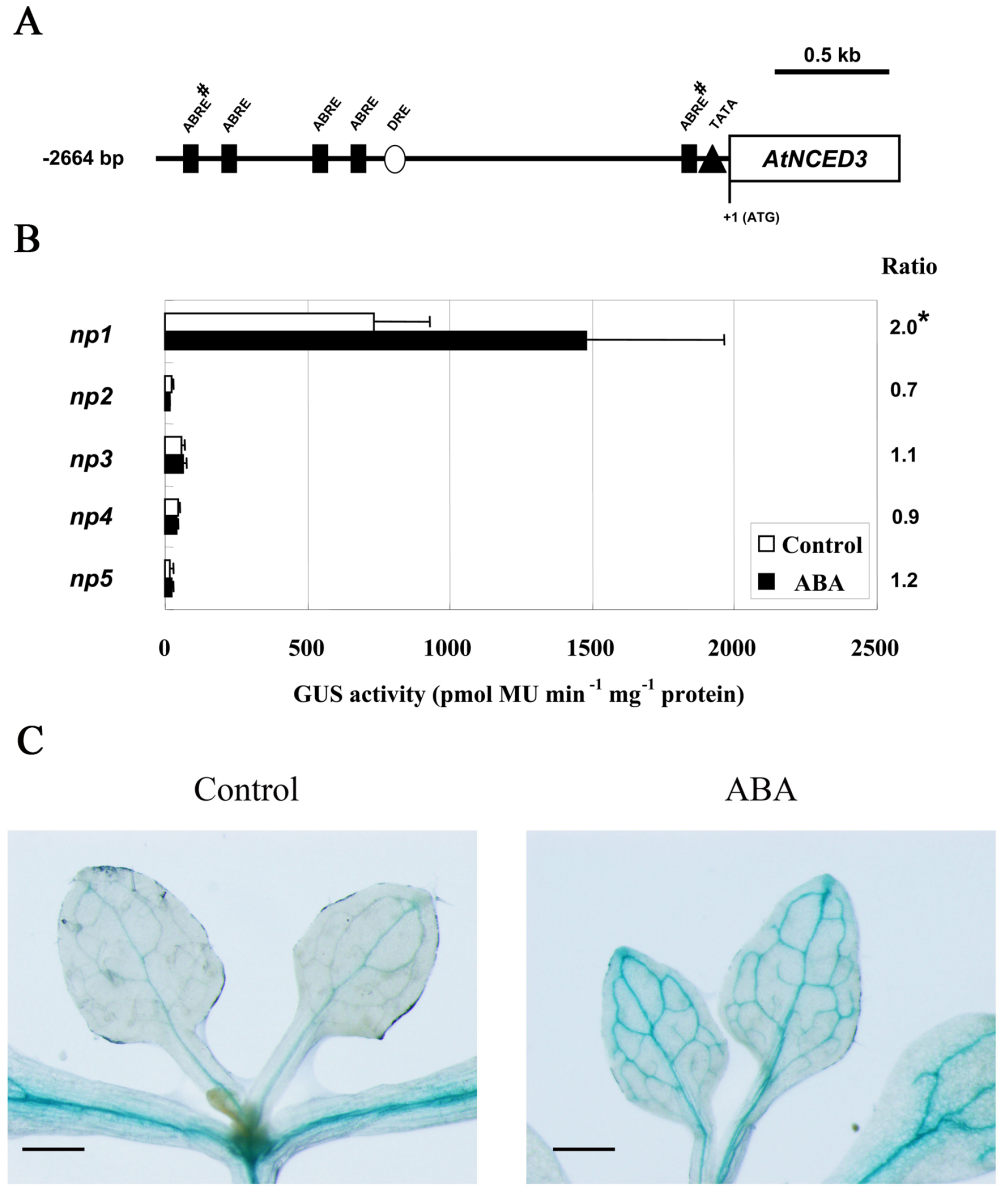
The 2664 bp region of *AtNCED3* promoter contains ABA responsive *cis*-element. (A) The analysis of putative *cis*-elements in 2664 bp *AtNCED3* promoter by Plant *Cis*-Acting Regulatory Element (PlantCARE) database. The A of the ATG codon at the translational site is defined as +1. Octothorpes indicate the ABREs with conserved core sequence. (B) GUS activities of transgenic plants for the 5’-deletion constructs. Values represent means of the activities of ten independent lines. Error bars represent the SD of three independent measurements. Data were analyzed by Student's t-test (*P<0.05; **P<0.01). The ratios indicate the fold change increases of the GUS activities after ABA treatment compared with values obtained from control treatment. (C) Histochemical analysis of GUS expression in the 2664 bp *AtNCED3* promoter-GUS transgenic plants with or without 100 µM ABA treatment for 5 h (scale bars represent 2.5 mm). 10 independent lines and 15 individual plants from each line were analyzed.

The *in silico* analysis of the promoter sequence of *AtNCED3* revealed the presence of five hypothetical ABREs [39]. In the five predicted ABREs, two were conserved in core sequence (CACGTG), one proximal (CACGTGGC, –206 to –198 bp) and one distal (GGCACGTG, –2372 to –2364 bp). A putative TATA box (TATATA) was located between –155 and –149 bp (Figure 2A).

In order to identify regions containing ABA responsive *cis*-element, at least 10 independent transgenic lines from each construct were sprayed with 100 µm ABA and analyzed after 5 h. The *np1* transgenic plants with the 2664 bp *AtNCED3* promoter exhibited a 2.0-fold increase in GUS activity when treated with ABA, whereas no induction was detected in transgenic plants harboring shorter promoter regions (Figure 2B). GUS staining showed elevated expression in vascular tissue of true leaves in *np1* transgenic plants after exogenous ABA application (Figure 2C). These data indicate that the 2664 bp region of *AtNCED3* promoter from the translation start site confers the ABA responsive expression of *AtNCED3* and the ABA responsive *cis*-element is located between the region –2664 and –2018 bp from the translation start site.

### A 87 bp region of *AtNCED3* promoter contains ABA responsive *cis*-element

**Figure 3:**
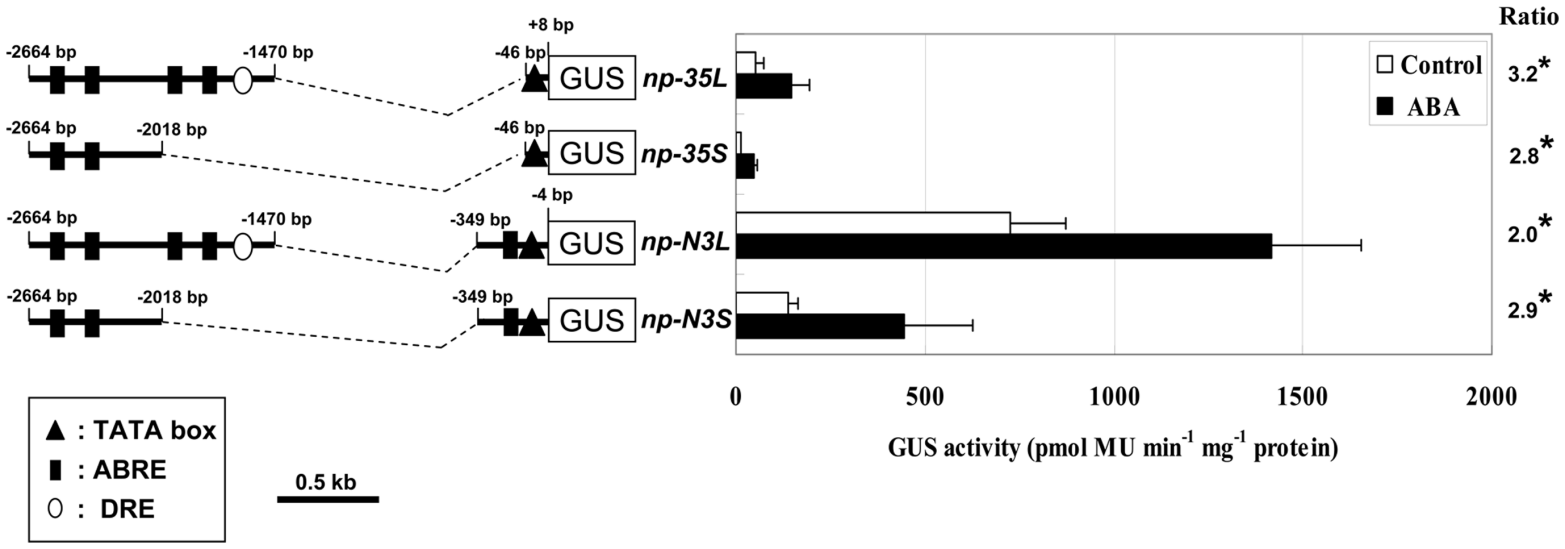
The region between -2664 and -2018 bp of *AtNCED3* promoter contains ABA responsive *cis*-element. Left, the different 3′-deletion constructs of the *AtNCED3* promoter-GUS. In constructs *np-35L* and *np-35S*, –2664 to –1470 bp or –2664 to –2018 bp fragments are fused to a CaMV 35S minimal promoter (–46 to +8 bp from transcription start site) respectively, followed by a GUS reporter gene. In constructs *np-N3L* and *np-N3S*, –2664 to –1470 bp and –2664 to –2018 bp fragments are fused to –349 bp *AtNCED3* promoter (–349 to –4 bp) respectively, followed by a GUS reporter gene. Right, GUS activities of transgenic plants for the 3′-deletion constructs are shown. Values represent means of the activities of ten independent lines. Error bars represent the SD of three independent measurements. Data were analyzed by Student's t-test (*P<0.05; **P<0.01). The ratios indicate the fold change increases of the GUS activities after ABA treatment compared with values obtained from control treatment.

To further confirm that the ABA responsive *cis*-element is located in the region between –2664 and –2018 bp, we employed a deletion-fusion approach. The 3′-deletion fragment (–2664 to –2018 bp) was fused to either a CaMV 35S minimal promoter (–46 to +8 bp from transcription start site) [40] or a –349 bp *AtNCED3* promoter (Figure 3). To rule out the possibility that one *cis*-element pairs with distal ones to establish a functional unit, the longer fragment (–2664 to –1470 bp) was ligated to a CaMV 35S minimal promoter and a –349 bp *AtNCED3* promoter, respectively (Figure 3). The –349 bp *AtNCED3* promoter has been demonstrated not in response to ABA (Figure 2B, *np5*). GUS activity driven by the –349 bp *AtNCED3* promoter was stronger than the CaMV 35S minimal promoter either with or without ABA treatment (Figure 3). GUS activity assay showed that all the transgenic plants harboring four different constructs displayed higher GUS activity after sprayed with 100 µm ABA (Figure 3). These results further confirm that the 546 bp region between –2664 and –2018 bp contains the *cis*-element responsive to ABA.

**Figure 4:**
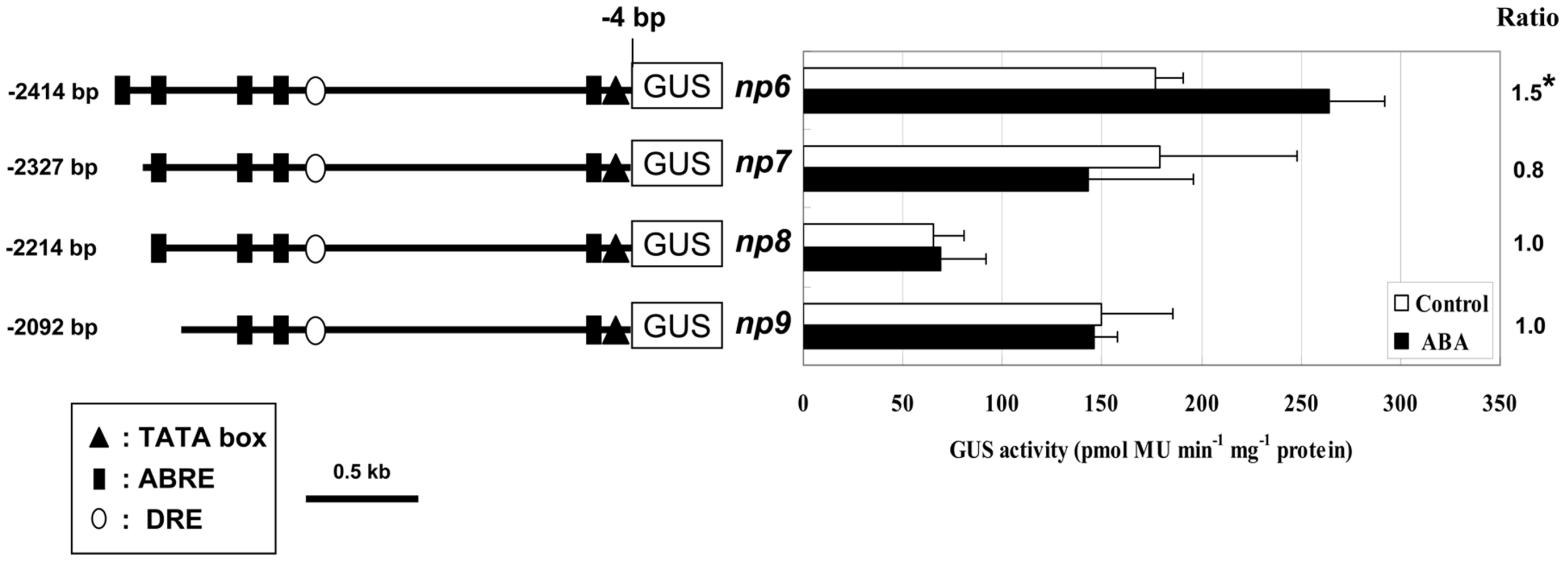
The region between–2414 and –2092 bp of *AtNCED3* promoter contains ABA responsive *cis*-element. Left, the different 5’-deletion constructs of the *AtNCED3* promoter between –2414 and –2092 bp region. The *AtNCED3* promoter regions between –2414 to –4 bp, –2327 to –4 bp, –2214 to –4 bp or –2092 to –4 bp are cloned upstream of GUS coding region. Right, GUS activities of transgenic plants for the 5′-deletion constructs are shown. Values represent means of the activities of ten independent lines. Error bars represent the SD of three independent measurements. Data were analyzed by Student's t-test (*P<0.05; **P<0.01). The ratios indicate the fold change increases of the GUS activities after ABA treatment compared with values obtained from control treatment.

To further delineate the region between –2664 and –2018 bp of the *AtNCED3* promoter, additional 5’ deletion constructs containing the regions –2414 to –4 bp, –2327 to –4 bp, –2214 to –4 bp or –2092 to –4 bp were made, and the corresponding transgenic plants were generated (Figure 4, *np6-np9*). When treated with ABA, the plants containing the 2414 bp promoter-GUS (*np6*) transgene showed a 1.5-fold increase in GUS expression; whereas, those containing promoters shorter than 2414 bp (*np7*, *np8* and *np9*) did not show any ABA induction (Figure 4). These results indicate that the putative ABA responsive *cis*-element is located in the 87 bp region between –2414 and –2327 bp from the translation start site of the *AtNCED3* promoter.

### An ABRE in 87 bp region is required for ABA induced expression of *AtNCED3*

**Figure 5:**
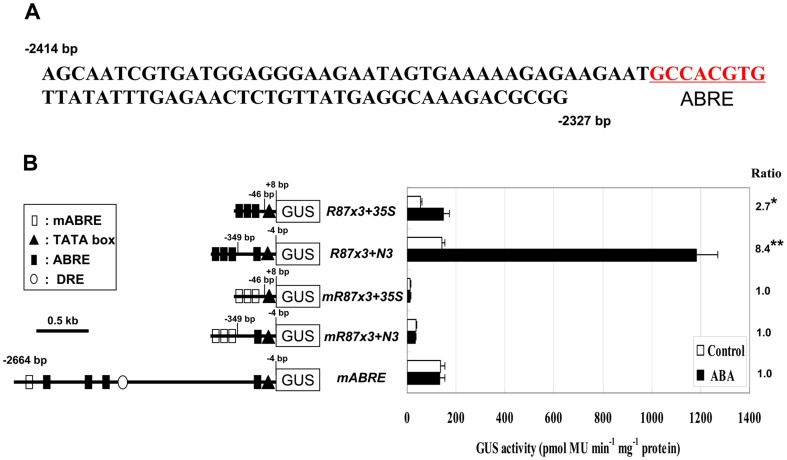
The distal ABRE of the *AtNCED3* promoter is involved in ABA-responsive expression. (A) The nucleotide sequence of the 87 bp region (–2414 to –2327 bp) in *AtNCED3* promoter. The ABRE motif marked in red. (B) Left, in constructs *R87x3+35S* and *R87x3+N3*, three tandem repeats of 87 bp region are fused to CaMV 35S minimal promoter and –349 bp *AtNCED3* promoter respectively, followed by a GUS reporter gene. In constructs *mR87x3+35S* and *mR87x3+N3*, three tandem repeats of mutated 87 bp region (GGCACGTG changed to TTCCGGGG ) are fused to CaMV 35S minimal promoter and –349 bp *AtNCED3* promoter respectively, followed by a GUS reporter gene. In construct *mABRE*, a –2664 bp *AtNCED3* promoter with base substitution at ABRE (GGCACGTG changed to TTCCGGGG between –2372 to –2364 bp) is fused to GUS reporter gene. Right, GUS activities of transgenic plants for the constructs are shown. Values represent means of the activities of ten independent lines. Error bars represent the SD of three independent measurements. Data were analyzed by Student's t-test (*P<0.05; **P<0.01). The ratios indicate the fold change increases of the GUS activities after ABA treatment compared with values obtained from control treatment.

The analysis of this 87 bp region identified a putative ABRE (Figure 5A). Thus, we tested whether this putative ABRE mediates the ABA responsive expression of *AtNCED3*. To do so, the region containing the putative ABA responsive *cis*-element, between –2414 and –2327 bp, was duplicated 3 times and then fused with either a CaMV 35S minimal promoter or –349 bp *AtNCED3* promoter, followed by GUS gene and named *R87x3-35S* and *R87x3-N3* (Figure 5B). A mutant version (*mR87x3-35S* and *mR87x3-N3*), in which the putative ABRE (GGCACGTG) was mutated to TTCCGGGG, was constructed in the same way to identify the function of this *cis*-element in response to ABA (Figure 5B). At the same time, we introduced base substitutions into the putative ABRE in –2664 bp *AtNCED3* promoter and named *mABRE* (Figure 5B). All constructs were transformed into Arabidopsis and transgenic plants were sprayed with 100 µm ABA. GUS activity assay showed that GUS activities were induced in *R87x3-35S* and *R87x3-N3* lines (2.7- and 8.4-folds, respectively), but not in *mR87x3-35S*, *mR87x3-N3* and *mABRE* lines (Figure 5B). The results indicate that the putative ABRE (GGCACGTG) is a functional ABA responsive *cis*-element in *AtNCED3* promoter, and GUS expression driven by -349 bp *AtNCED3* promoter is stronger than that by the CaMV 35S minimal promoter with or without ABA treatment.

### *AtNCED3* expression is not transactivated by ABF3

**Figure 6:**
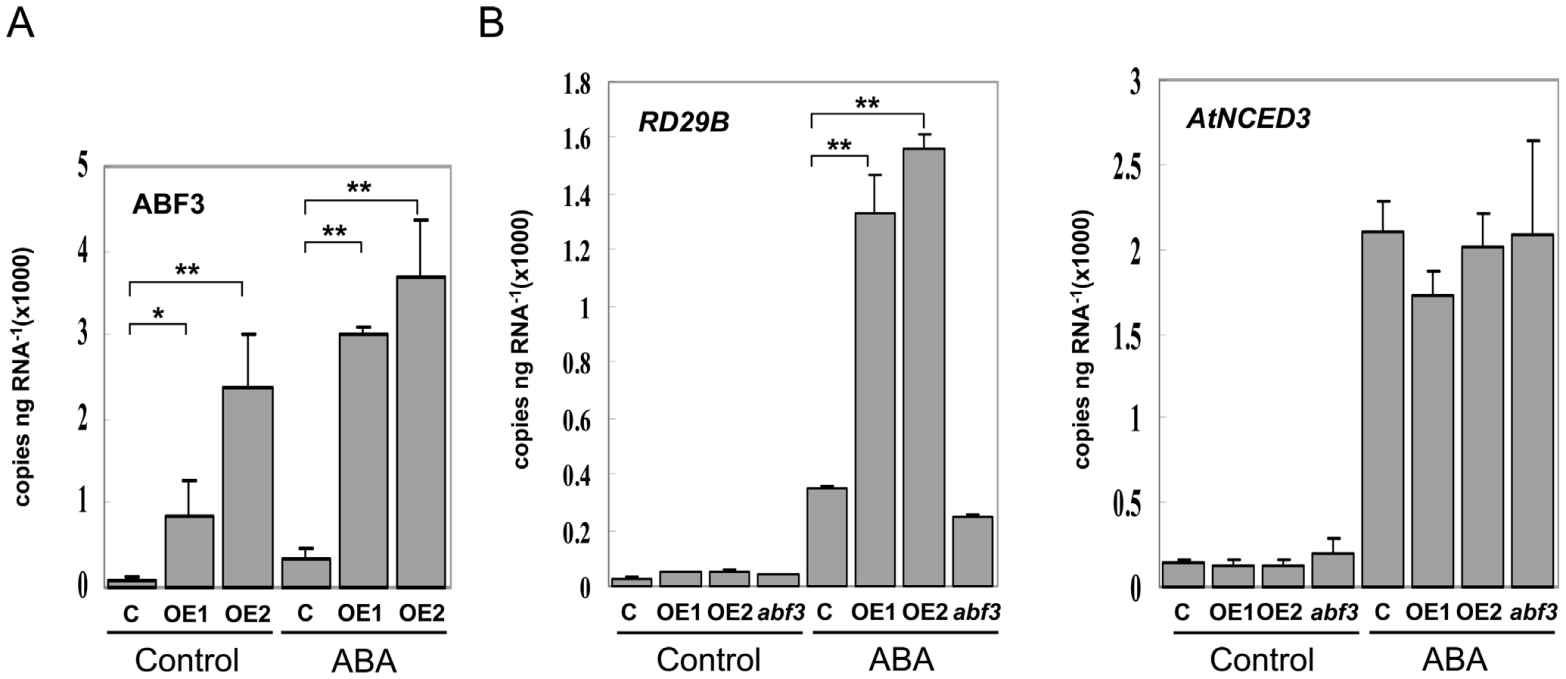
The expression of *AtNCED3* in WT, ABF3 overexpression transgenic plants (OE) and *abf3*. (A) Expression of the ABF3 assayed by quantitative RT-PCR in Col-0 (C), ABF3 OE1 (OE1) and ABF3 OE2 (OE2) two-week-old whole seedlings under control conditions or after 5 h of exposure to 100 µM ABA. Data are means ± SE (n  = 3). Data were analyzed by Student's t-test (*P<0.05; **P<0.01). (B) Expression of the *RD29B* and *AtNCED3* assayed by quantitative RT-PCR in Col-0 (C), ABF3 OE1 (OE1), ABF3 OE2 (OE2) and ABF3 mutant (*abf3*) two-week-old whole seedlings under control conditions or after 5 h of exposure to 100 µM ABA. Data are means ± SE (n  = 3). Data were analyzed by Student's t-test (*P<0.05; **P<0.01).

Previous studies showed that ABF3 can recognize ABRE and that its expression was induced by ABA and dehydration stress, providing a possibility that *AtNCED3* expression is directly regulated by ABF3 [26], [27]. To test this possibility, we examined the expression of *AtNCED3* in both ABF3 over-expression (OE) transgenic plants and ABF3 mutant (*abf3*). The *abf3* mutant (SALK_096965) was proven to be a null mutant with a T-DNA insertion in intron 2 [41]. The ABF3-OE lines produced several times expression of ABF3 (Acc. 001036708) (Figure 6A). Quantitative RT-PCR analysis showed that application of 100 µM ABA induced *AtNCED3* expression in control and ABF3-OE plants (Figure 6B). However, its expression in the ABF3-OE plants was similar to that observed in the control plants, suggesting that over-expression of ABF3 did not enhance the ABA-induced *AtNCED3* expression (Figure 6B). Consistent with this result, loss of ABF3 function in the *abf3* mutant did not affect *AtNCED3* expression either with or without ABA treatment (Figure 6B). An ABF3 regulated gene, *RD29B* (Acc. D13044), was used as positive control. These results together indicate that *AtNCED3* expression is not transactivated by ABF3.

## Discussion

Significant progress has been made in understanding ABA perception and downstream gene activation mechanisms when plants encounter stresses [42], [43]. The mechanism by which plants perceive stress and initiate *de novo* synthesis of ABA is obscure, although stress-induced ABA synthesis has been known since 1960’s [44]. Stress-induced ABA accumulations can be ABA-dependent or -independent [22], [45]. But the molecular basis for such accumulation is poorly understood. In Arabidopsis, AtNCED3 is the key step regulating ABA biosynthesis. *AtNCED3* expression is highly induced by various stresses leading to enhanced ABA biosynthesis [45]. By analyzing a series of delineated promoters of *AtNCED3*, coupled with mutagenesis, we revealed that a distal ABRE (–2372 to –2364 bp) is critical for the ABA induced *AtNCED3* expression. This element escaped detection in earlier reports where promoters shorter than 1.5 kb were used in promoter-reporter analysis [33]. Indeed, these constructs failed to show an expression pattern consistent with the endogenous *AtNCED3*, particularly the expression in vascular tissues of leaves and stress induction [46]. The identification of this distal element in *AtNCED3* promoter provides the molecular basis for the ABA-dependent ABA accumulation.

Recently Behnam et al [47] reported that the same distal ABRE is required for dehydration induction of *AtNCED3* expression. Thus, this element is critical to ABA and dehydration induced *AtNCED3* expression, hence ABA accumulation in plants under stresses. This raises the question whether the activation of *AtNCED3* expression by ABA and dehydration involved identical or different mechanisms. In one scenario, dehydration may increase *AtNCED3* expression by an ABA-independent manner; then the increased ABA triggers further increase in *AtNCED3* expression in an ABA-dependent manner. As a result, an increase of ABA content is achieved quickly after the plant encounters stresses. In another scenario, dehydration may increase locally ABA content independently from *AtNCED3* expression, but via hydrolysis of ABA-GE to produce active ABA [48]. The accumulation of ABA induces/stimulates *AtNCED3* expression which causes further increase of ABA. It can also be argued that the application of ABA is perceived as a stress by the plant; hence it initiates stress response, which leads to increased ABA biosynthesis. However, this interpretation is not in agreement with the reduction of *AtNCED3* expression in ABA-deficient mutants under stresses [7], [14]. In any cases, this distal ABRE element provides the molecular basis for ABA/stress regulation of *AtNCED3* expression. This information can be used to identify the transcription factors that directly regulate *AtNCED3* transcription, which may be an entry point to dissect the signaling pathway from stress perception to ABA synthesis.

**Figure 7:**
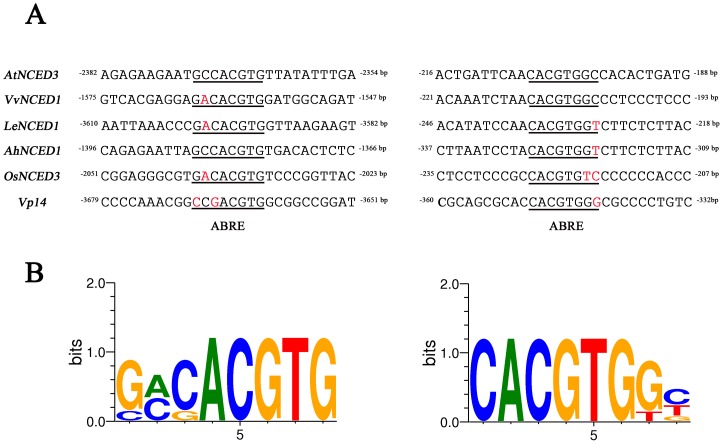
The distal and proximal ABREs are conserved in drought induced *NCED* promoters. (A) Comparison of the conserved ABRE in the distal region and proximal region of drought induced *NCED* promoters from Arabidopsis (*AtNCED3*; Acc. NM_112304), grape (*VvNCED1*; Acc. AY337613), tomato (*LeNCED1*; Acc. Z97215), peanut (*AhNCED1*; Acc. EU497940), rice (*OsNCED3*; Acc. AY838899) and maize (*Vp14*; Acc. U95953). Red letter represents non-conserved nucleotide. (B) Sequence logos for distal ABRE (left) and proximal ABRE (right). Sequence logos were created online using the Weblogo resource [59].

Many drought-induced *NCEDs* have been studied. By comparing *AtNCED3* promoter with the drought-induced *NCED* promoters from grape (*Vitis vinifera*) [49], tomato [18], peanut (*Arachis hypogaea* L.) [50], rice (*Oryza sativa*) [51] and maize [9], a conserved 6 nucleotide core (C/GACGTG) emerged as a distal ABA responsive *cis*-element (Figure 7A, B). Two of them, the distal ABRE in *AhNCED1* promoter (–1386 to –1379 bp) and *AtNCED3* promoter (–2372 to –2364 bp) have been identified to respond to drought [47], [50], suggesting the ABRE from different species may have the similar function. However, there is no evidence whether the distal ABA responsive *cis*-elements from other species are involved in drought responsive transcription. Further studies will be needed to identify whether these ABREs contribute to ABA response.

Functional ABREs are frequently found close to transcription start site. Two ABREs for ABA induction of *HVA22* expression in barley are located between –240 and –84 bp of the *HVA22* promoter from transcription start site [28]. An ABRE required for ABA responsive expression of the *rd29A* in Arabidopsis is located between –63 to –55 bp transcription start site [30]. Similar cases were found in the *MIR168a* in Arabidopsis (–126 to –122 bp from transcription start site) and *Vp1* in maize (–84 to –75 bp from transcription start site) [31], [52]. In the promoter of Arabidopsis and other five species, we found a putative ABRE (CACGTG) near the transcription start site (Figure 7A, B), but this proximal putative ABRE in Arabidopsis was not necessary for ABA induced *AtNCED3* expression. Firstly, progressive deletion of the promoter sequences up to 2327 bp position did not detect ABA responsive expression of the reporter gene (Figure 4A, *np6*). Secondly, the –2664 to –2018 bp or –2664 to –1470 bp region fused to minimal CaMV 35S promoter is sufficient for ABA induced expression of *AtNCED3* (Figure 3A, *np-35L* and *np-35S*). These data indicate that the proximal putative ABRE is not necessary for ABA response. However, we noted that any promoter fragments fused to –349 bp *AtNCED3* promoter containing the proximal putative ABRE can enhance the response to ABA (Figure 3A, *np-N3L* and *np-N3S*; Figure 5A, *R* *87x3-N3* and *mR87x3-N3*), and the –349 bp *AtNCED3* promoter was enough for *AtNCED3* expression in early development stage (Figure 1A, E, G and O), suggesting that this proximal ABRE may possess enhancer function or confer specific *AtNCED3* expression in response to developmental cues.

The 1.5 kb promoter-GUS failed to confer stress induced GUS expression although it is known that *AtNCED3* is strongly induced in stressed leaves [33]. The 1 kb promoter, on the other hand, delivered stress induced expression of LUC [37], [38]. However, this study and others [47] convincingly demonstrated that the *cis*-elements responsible for ABA and stress induction are located on the distal end of the *AtNCED3* promoter. Besides the possibility of repressors in the ∼500 bp (1 to 1.5 kb) region, we tend to believe that detection methods are responsible for the discrepancy. Detection of LUC is much more sensitive than GUS staining. Based on existing data, we think that the distal *cis*-elements defined by this study play a predominant role in stress induced *AtNCED3* expression, whereas the proximal ones (such as the ones included in the 1 kb promoter) may play a minor role. Conceivably even with a 1 kb promoter, there will be many genes that can affect the promoter activity, either directly or indirectly. In fact, the cuticle synthetic gene reflects an indirect regulation on *AtNCED3* expression [37].

It is unexpected to find that ABF3 can not activate the expression of *AtNCED3*, in view of that ABF3 is known to recognize ABRE and activates ABA responsive genes. Indeed, ABRE is very similar to the G box (CACGTG) which is involved in the red light signaling [53], jasmonic acid response [54], and alicylic acid response [55], suggesting that different transcription activators can recognize the core sequence (CACGTG). In addition, transcriptome analysis of ABF3 over-expression transgenic Arabidopsis demonstrated that *AtNCED3* was regulated similarly in over-expression and control plant lines under drought treatment [56]. We are, however, somewhat careful about the conclusion of ABF3 can not activate *AtNCED3* given that three AREB/ABF transcription factors (AREB1, AREB2, ABF3) are functionally redundant [41]. Thus, it would be more convincing to test the expression of *AtNCED3* in *areb1 areb2 abf3* triple mutant.

**Figure 8:**
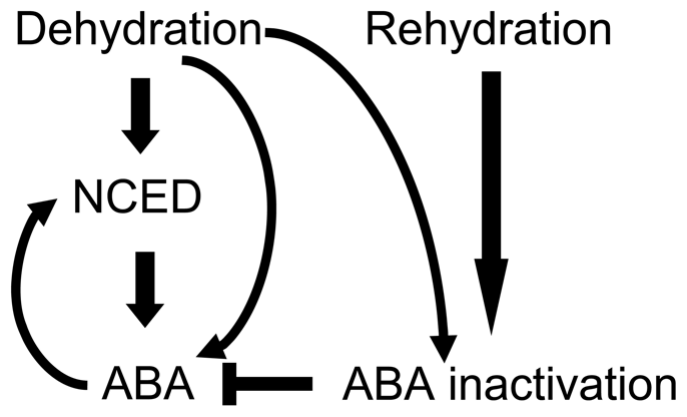
Schematic representation of the balance between ABA biosynthesis and inactivation under different conditions. When plants encounter drought stress, ABA is accumulated by de novo synthesis or activation of inactive ABA pool (ABA-GE hydrolysis); then further increased by positive feedback biosynthetic pathway. ABA inactivation pathway is also activated by drought stress to maintain endogenous ABA levels within the permissible range. Once stress is relieved, ABA inactivation pathway allows for rapid degradation of ABA to reach a new balance between ABA biosynthesis and inactivation. Arrow lines indicate stimulation of gene expression, ABA production or ABA inactivation pathway whereas a line with stopper bar represents reduction of ABA content.

The positive feedback regulation on *AtNCED3* expression by ABA raises a question on how the plant maintains its ABA levels under different conditions. We propose a model to try to explain it (Figure 8). When a plant encounters drought stress, ABA biosynthesis is activated [17], and interestingly the ABA hydroxylation pathway is also induced [57], [58]. ABA is inactivated by the hydroxylation pathway and the conjugation pathway [5], and the former is the predominant ABA inactivation pathway [4]. When the stress is relieved, ABA quickly returns to a normal level. Thus, we hypothesize that the ABA level is maintained by the balance between ABA synthesis and ABA inactivation activities. It is possible that the hydroxylation pathway is highly active, which plays a major role in maintaining endogenous ABA levels within the permissible range. This notion is supported by the quick reduction of ABA when stresses are relieved.

## Supporting Information

Table S1 **Primers used in this study.**
(PPT)
