# Supplementary material for: A Distal ABA Responsive Element in AtNCED3 Promoter Is Required for Positive Feedback Regulation of ABA Biosynthesis in Arabidopsis
Source: PLoS One. 2014 Jan 27;9(1):e87283. doi: 10.1371/journal.pone.0087283 (PMC3903620; doi:10.1371/journal.pone.0087283)
Supplement: Table S1 — Primers used in this study. (PPT) [file pone.0087283.s001.ppt]

## Slide 1
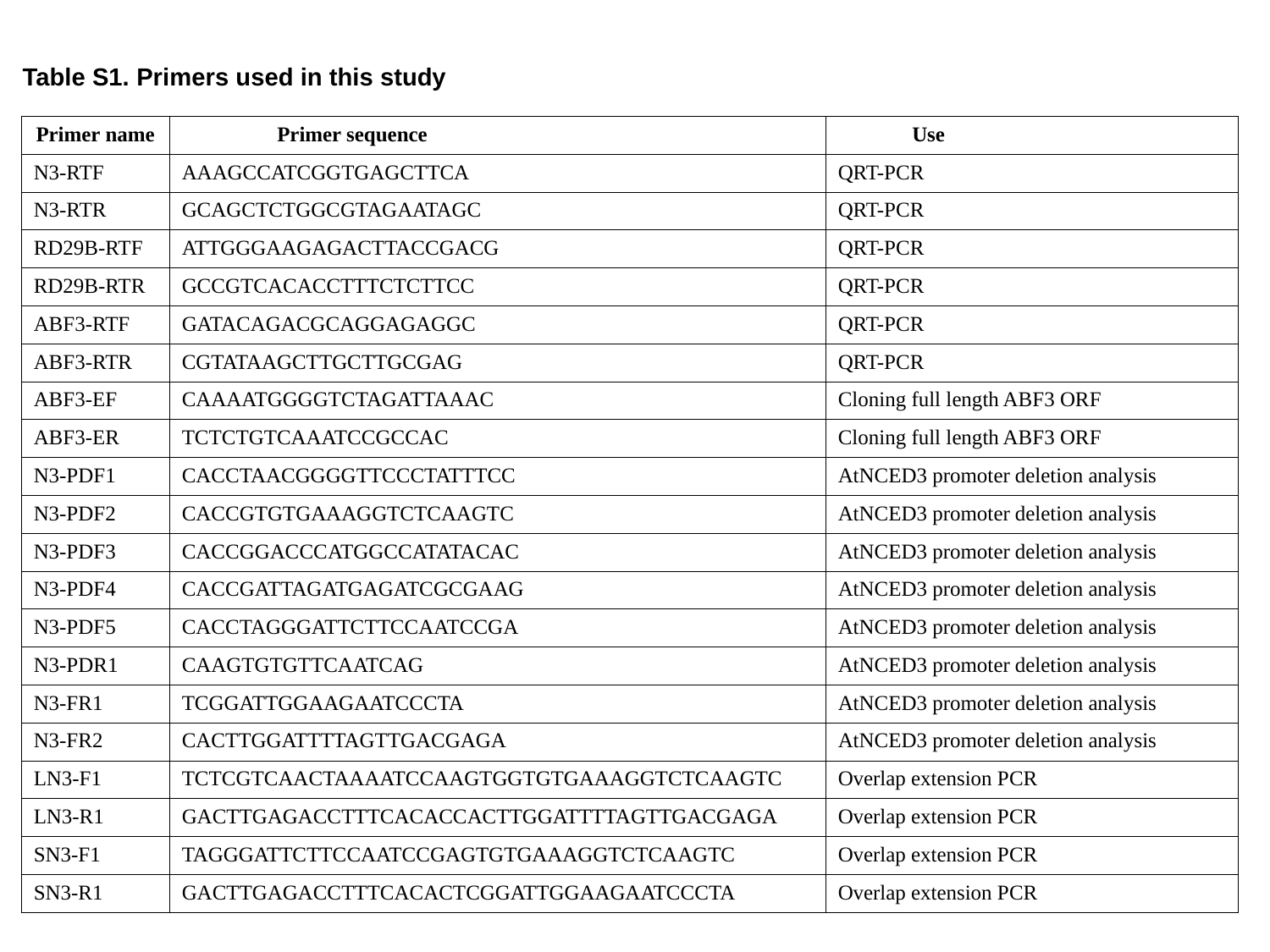

Table S1. Primers used in this study
| Primer name | Primer sequence | Use |
| --- | --- | --- |
| N3-RTF | AAAGCCATCGGTGAGCTTCA | QRT-PCR |
| N3-RTR | GCAGCTCTGGCGTAGAATAGC | QRT-PCR |
| RD29B-RTF | ATTGGGAAGAGACTTACCGACG | QRT-PCR |
| RD29B-RTR | GCCGTCACACCTTTCTCTTCC | QRT-PCR |
| ABF3-RTF | GATACAGACGCAGGAGAGGC | QRT-PCR |
| ABF3-RTR | CGTATAAGCTTGCTTGCGAG | QRT-PCR |
| ABF3-EF | CAAAATGGGGTCTAGATTAAAC | Cloning full length ABF3 ORF |
| ABF3-ER | TCTCTGTCAAATCCGCCAC | Cloning full length ABF3 ORF |
| N3-PDF1 | CACCTAACGGGGTTCCCTATTTCC | AtNCED3 promoter deletion analysis |
| N3-PDF2 | CACCGTGTGAAAGGTCTCAAGTC | AtNCED3 promoter deletion analysis |
| N3-PDF3 | CACCGGACCCATGGCCATATACAC | AtNCED3 promoter deletion analysis |
| N3-PDF4 | CACCGATTAGATGAGATCGCGAAG | AtNCED3 promoter deletion analysis |
| N3-PDF5 | CACCTAGGGATTCTTCCAATCCGA | AtNCED3 promoter deletion analysis |
| N3-PDR1 | CAAGTGTGTTCAATCAG | AtNCED3 promoter deletion analysis |
| N3-FR1 | TCGGATTGGAAGAATCCCTA | AtNCED3 promoter deletion analysis |
| N3-FR2 | CACTTGGATTTTAGTTGACGAGA | AtNCED3 promoter deletion analysis |
| LN3-F1 | TCTCGTCAACTAAAATCCAAGTGGTGTGAAAGGTCTCAAGTC | Overlap extension PCR |
| LN3-R1 | GACTTGAGACCTTTCACACCACTTGGATTTTAGTTGACGAGA | Overlap extension PCR |
| SN3-F1 | TAGGGATTCTTCCAATCCGAGTGTGAAAGGTCTCAAGTC | Overlap extension PCR |
| SN3-R1 | GACTTGAGACCTTTCACACTCGGATTGGAAGAATCCCTA | Overlap extension PCR |

## Slide 2
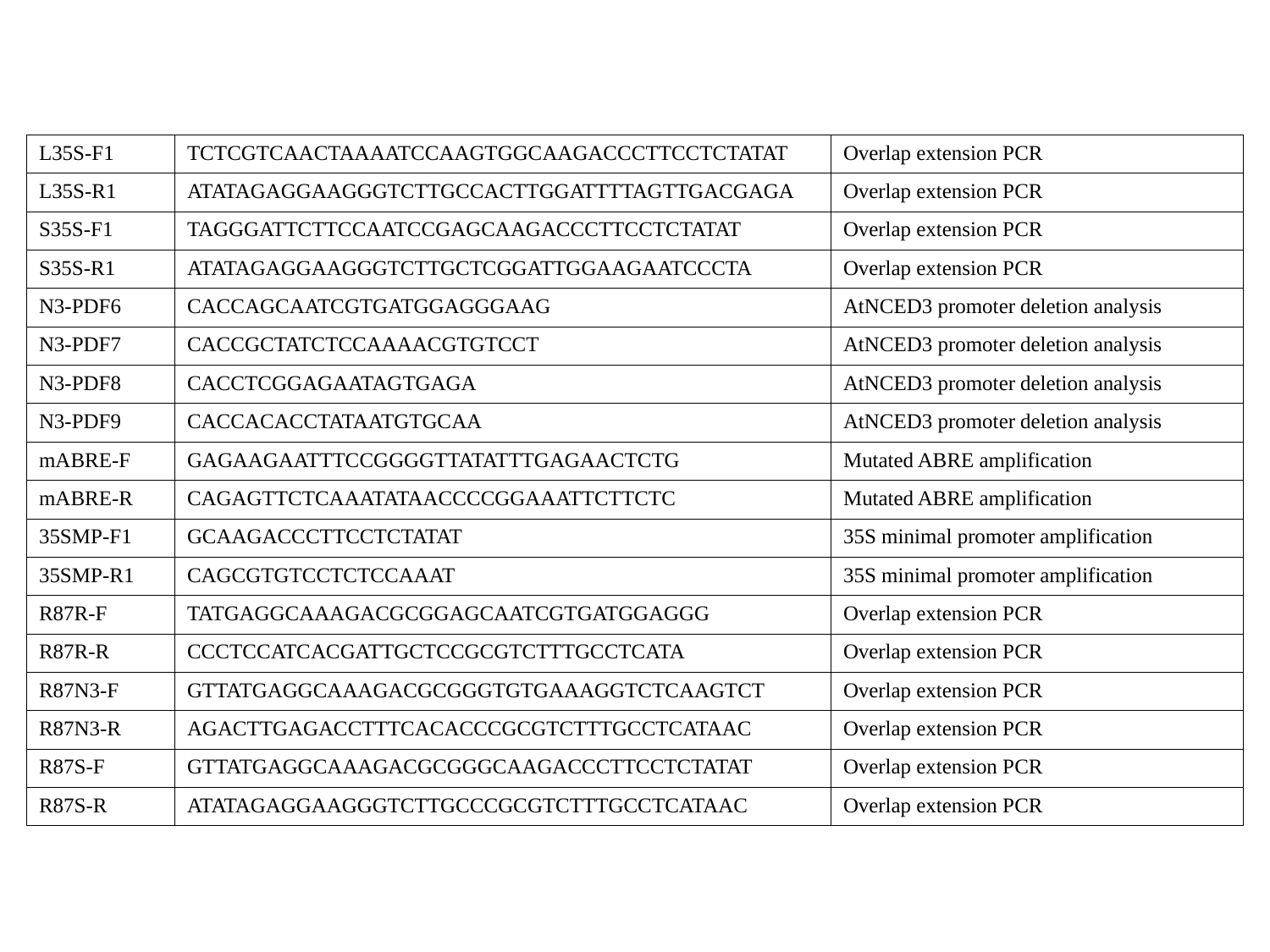

| L35S-F1 | TCTCGTCAACTAAAATCCAAGTGGCAAGACCCTTCCTCTATAT | Overlap extension PCR |
| --- | --- | --- |
| L35S-R1 | ATATAGAGGAAGGGTCTTGCCACTTGGATTTTAGTTGACGAGA | Overlap extension PCR |
| S35S-F1 | TAGGGATTCTTCCAATCCGAGCAAGACCCTTCCTCTATAT | Overlap extension PCR |
| S35S-R1 | ATATAGAGGAAGGGTCTTGCTCGGATTGGAAGAATCCCTA | Overlap extension PCR |
| N3-PDF6 | CACCAGCAATCGTGATGGAGGGAAG | AtNCED3 promoter deletion analysis |
| N3-PDF7 | CACCGCTATCTCCAAAACGTGTCCT | AtNCED3 promoter deletion analysis |
| N3-PDF8 | CACCTCGGAGAATAGTGAGA | AtNCED3 promoter deletion analysis |
| N3-PDF9 | CACCACACCTATAATGTGCAA | AtNCED3 promoter deletion analysis |
| mABRE-F | GAGAAGAATTTCCGGGGTTATATTTGAGAACTCTG | Mutated ABRE amplification |
| mABRE-R | CAGAGTTCTCAAATATAACCCCGGAAATTCTTCTC | Mutated ABRE amplification |
| 35SMP-F1 | GCAAGACCCTTCCTCTATAT | 35S minimal promoter amplification |
| 35SMP-R1 | CAGCGTGTCCTCTCCAAAT | 35S minimal promoter amplification |
| R87R-F | TATGAGGCAAAGACGCGGAGCAATCGTGATGGAGGG | Overlap extension PCR |
| R87R-R | CCCTCCATCACGATTGCTCCGCGTCTTTGCCTCATA | Overlap extension PCR |
| R87N3-F | GTTATGAGGCAAAGACGCGGGTGTGAAAGGTCTCAAGTCT | Overlap extension PCR |
| R87N3-R | AGACTTGAGACCTTTCACACCCGCGTCTTTGCCTCATAAC | Overlap extension PCR |
| R87S-F | GTTATGAGGCAAAGACGCGGGCAAGACCCTTCCTCTATAT | Overlap extension PCR |
| R87S-R | ATATAGAGGAAGGGTCTTGCCCGCGTCTTTGCCTCATAAC | Overlap extension PCR |
